# Novel duplication on chromosome 16 (q12.1-q21) associated with behavioral disorder, mild cognitive impairment, speech delay, and dysmorphic features: case report

**DOI:** 10.3325/cmj.2011.52.415

**Published:** 2011-06

**Authors:** Ljubica Odak, Ingeborg Barišić, Leona Morožin Pohovski, Mariluce Riegel, Albert Schinzel

**Affiliations:** 1 Children's Hospital Zagreb, Clinical Hospital Center Sisters of Mercy Zagreb, Zagreb, Croatia; 2 Institute of Medical Genetics University of Zürich, Zürich, Switzerland

## Abstract

We report on the 10-year follow-up and clinical, cytogenetic, and molecular investigation of a girl admitted for evaluation because of speech delay, learning difficulties, aggressive behavior, and dysmorphic facial features that included high forehead, round face, epicanthic folds, low-set dysplastic ears, flat nasal bridge, long flat philtrum, thin upper lip, small mouth, and short neck. The analysis of high-resolution GTG- and CTG-banding chromosomes suggested a de novo direct duplication of 16q12-q21 region and fluorescence in situ hybridization analysis with whole-chromosome specific 16 probe confirmed that the duplicated genetic material originated from the chromosome 16. Subsequently, array-based comparative genomic hybridization analysis with a ≈ 75 kb resolution showed a 9.92 Mb gain on the long arm of chromosome 16 at bands q12.1 through q21. To the best of our knowledge, this is the first case of duplication 16q12.1q21 described in literature. Several genes within the duplicated region are possibly correlated with clinical features present in our patient. Clinical and cytogenetic findings were compared with the small number of reported patients with pure duplications 16q, partially overlapping the one in our patient. Clinical phenotype seems to be distinctive between the proximal-intermediate and intermediate-distal regions of the long arm of the chromosome 16. In particular, we observed a set of dysmorphic features that could present a characteristic dup 16q11.2-q13 phenotype. The present study illustrates the advantages of an integrative approach using both conventional and molecular techniques for the precise characterization and genotype-phenotype correlation in patients with dysmorphism, behavioral problems, and learning difficulties.

Non-mosaic full trisomy 16 is incompatible with survival and is commonly found in spontaneous abortions (1,2). Duplication of the entire long arm of the chromosome 16 is a rare disorder, often associated with various congenital anomalies, severe psychomotor retardation, and limited survival beyond childhood (3). Most of the recorded partial duplications of the long arm of the chromosome 16 result from the meiotic malsegregation of a parental balanced structural chromosomal rearrangement and are associated with deletions of different segments of other chromosomes, making genotype-phenotype correlation difficult.

Pure 16q duplications are rare (4-17). They are clinically important because they provide a basis for a better understanding of relationships between chromosomal abnormality and observed clinical manifestations. A literature review revealed 21 cases of a pure duplication of 16q spanning the region from q11 to q24. Fifteen cases (7 familiar) covered the proximal 16q11q13 region (4-11), 4 covered the proximal-intermediate 16q21-16q22 region (12-15), and only 2 covered a more distal q23-q24 region (16,17).

This article describes a girl with mild dysmorphic features, speech delay, learning disabilities, and behavioral problems. She carries a 9.92 Mb de novo tandem 16q12.1q21 duplication detected by high-resolution karyotype and further characterized by array comparative genomic hybridization (CGH). To the best of our knowledge, this is the first case of duplication 16q12.1-q21 described in literature.

## Methods

Conventional cytogenetic analysis on high-resolution GTG- and CTG-banded chromosomes was performed according to the standard technique on cultured lymphocytes from the father, mother, and proband. Whole chromosome-specific 16 painting probe (WCP 16) (Vysis, Downers Grove, IL, SAD) was used for fluorescence in situ hybridization (FISH) to metaphase the chromosomes. Hybridization, post hybridization washes, detection, and visualization of probes were performed according to standard procedures. Oligonucleotide array comparative genomic hybridization was carried out using test genomic DNA obtained with a standard salt extraction method. Agilent human CGH microarrays with a ~ 75 kb resolution (Agilent Human Genome Microarray Array-CGH 4x44K, customer array, design AMADID number 017457) was employed to perform further analysis according to the manufacturer's protocol.

## Results

### Clinical findings

A 3.5-year old girl was referred to our clinic with symptoms of speech delay, dysmorphic facial features, and aggressive behavior. Family history was unremarkable. Parents were healthy and unrelated, the mother was 38 and father was 36 years old. Her 5-year-old sister and 2-year old brother were healthy. The patient was born at 39 gestational weeks after an uncomplicated pregnancy. Birth weight was 3800 g (95th centile), length was 51 cm (75th centile), and occipito-frontal head circumference (OFC) was 34 cm (50th centile). Apgar score was 10 at both 5 and 10 minutes. Neonatal period was complicated with omphalitis, conjunctivitis, and respiratory and urinary tract infections. Because of the right hip dysplasia, she was treated with Pavlik harness. She sat at 9 months and started to walk at 13 months, but her speech was delayed. First words appeared at 16-18 months, and at the age of 3 years she was using only 30 words. During infancy and early childhood she had recurrent episodes of respiratory and urinary tract infections, but standard clinical follow-up did not disclose the presence of immune deficiency or structural abnormality of urinary or respiratory system.

**Figure 1:**
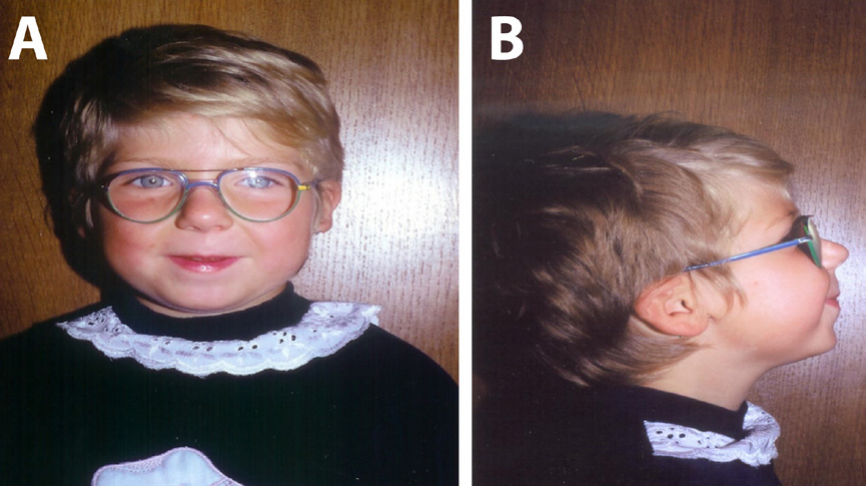
**(A, B).** The patient at 3 years and 5 months. Characteristics such as high forehead, round face, dysplastic and low-set ears, palpebral fissures slant down, flat nasal bridge, bulbous nose, long flat philtrum, thin upper lip, small mouth, small, slightly receding chin, and short neck are visible.

At the evaluation carried out at 3 years and 5 months, her height was 98 cm (50th centile), weight 16.9 kg (90th centile), and OFC 48.5 cm (50th centile). Physical examination revealed the presence of mild dysmorphic features including high forehead, round face, epicanthic folds, and low-set ears with poor folding of the helix, flat nasal bridge, long flat philtrum, thin upper lip, small mouth, slightly receding chin, and short neck. (Figure 1 A and B). She had stocky build and broad thorax. Hands and feet were small with stubby fingers. She was hyperactive with deficits in gross motor skills manifesting as insecurity in running, walking, and jumping, and showed poor visuospatial skills. Her behavior was aggressive with severe outbursts of anger in response to frustration and she tended to be dominant in interaction with other children. Auditory evaluation was normal. Ophthalmologic examination revealed strabismus and she was prescribed glasses for hypermetropia. Ultrasound examination of the abdomen, pelvis, and heart did not reveal structural anomalies. Electroencephalography and computerized tomography of the brain showed normal results. Metabolic screening and fragile X syndrome mutation analysis were negative. Her expressive speech was poor and she took part in occupational and speech therapy with significant improvement during childhood. Psychological assessments led to her receiving educational support for learning difficulties and emotional and behavioral problems while attending regular schooling. At the age of 11 years, she was transferred to a school for children with mild learning difficulties. At present, she is an 18-year-old adolescent. Her height is 165 cm (60th centile), weight 75 kg (>95th centile), and OFC 54.5cm (50th centile). She has mild learning difficulties, occasional temper tantrums, and difficulties in managing anger, but is otherwise free from health problems.

### Cytogenetic and molecular analysis

**Figure 2:**
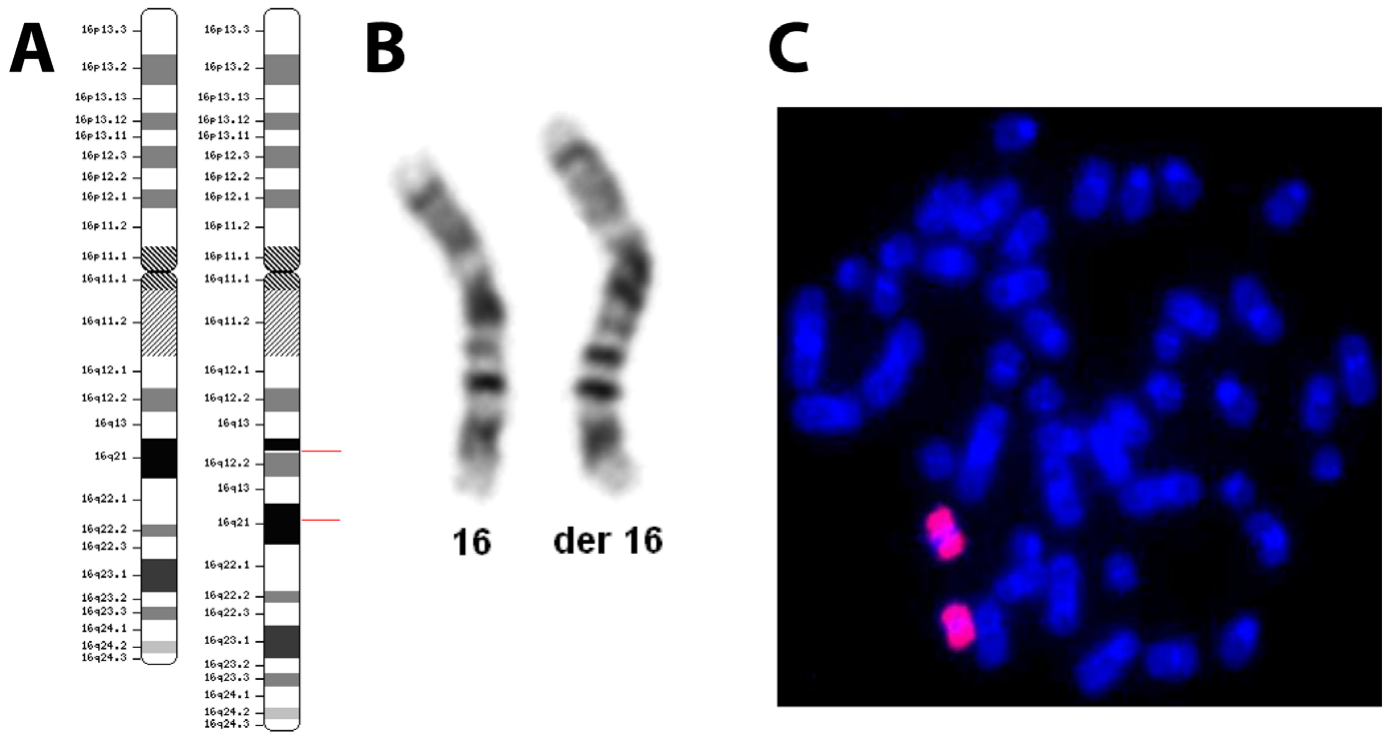
**(A)** Idiogram illustrating normal chromosome 16 and chromosome 16 with tandem duplication of the 16q12.1-q21 region (demarcated by lines). (**B**) High resolution karyotype, GTG banding showing the suspected interstitial dup16q13-q22. **(C)** Fluorescence in situ hybridization analysis using whole chromosome 16 painting probe on a metaphase of the proband demonstrated that both chromosomes 16 were completely colored.

**Figure 3:**
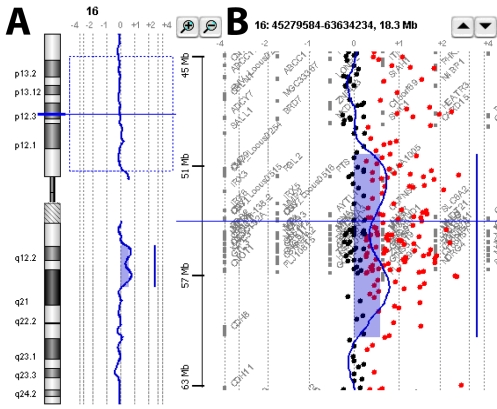
**(A)** Molecular characterization of the chromosome 16 of the proband using oligonucleotide array-based Comparative Genomic Hybridization (arrayCGH) showing a 9.92 Mb 16q12-1q21 duplication. The final result of the molecular analysis was defined as arr 16q12.1q21(50,843,158-60,770,795)x3 B. Enlarged view of the duplicated region (shadowed).

**Figure 4:**
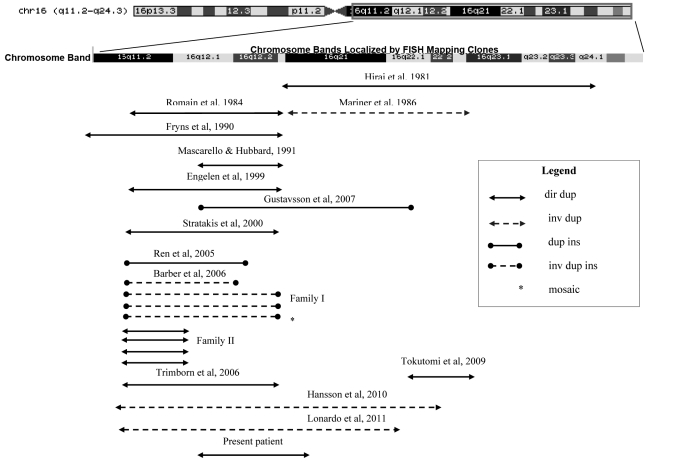
Map of the chromosome 16 showing the approximate location of the previously described duplications 16q and the one present in our patient. Direct duplications are represented by arrows and inverted duplications by dotted lines.

Cytogenetic analysis was performed on peripheral blood sample of the patient and her parents. Both parents presented with normal karyotypes while analysis of high-resolution GTG- and CTG-banded chromosomes suggested presumably direct duplication of 16q13q22 region of the proband (Figure 2A). FISH with WCP-16 stained the aberrant chromosome completely, confirming that the extra material was from the chromosome 16 (Figure 2B). The karyotype was defined as 46,XX,dup(16)(pter→ q22::q13→qter)dn. To characterize the duplicated region at a higher resolution and to exclude the possible additional cryptic rearrangements, oligonucleotide whole genome array CGH analysis was performed with a ~ 75 kb resolution array. The analysis showed partial duplication of the chromosome 16 involving 16q12.1-16q21 region, which is approximately 9.92 Mb in size. Map positions are based on the University of California Santa Cruz Genome Browser February 2009, gh19 (NCBI Build 37.1 reference sequence) (Figure 3). The final array result was 46,XX.arr 16q12.1q21(50,843,158-60,770,795)x3 dn. A map of the chromosome 16q with approximate size and location of the duplication found in our patient and of duplications previously described in literature is presented in Figure 4.

## Discussion

Complete duplication of 16q is associated with spontaneous abortion and affected individuals who survive till birth rarely live beyond childhood (3,18). Pure partial duplications of the long arm of the chromosome 16q are uncommon (Table 1). Clinical features observed in patients with pure partial duplication of various segments of 16q are summarized in Table 1. Although the number of reported cases is small, the classification in more consistent groups – proximal, proximal-intermediate, and intermediate-distal has been proposed in an effort to delineate the phenotypic spectrum for those particular chromosomal regions (15).

**Table 1:** Clinical findings in patients with partial duplications 16q reported in literature and in our patient. Plus designates the presence of a symptom and minus designates its absence

	Proximal duplications					Proximal- intermediate duplications					Intermediate- distal duplications	
**Duplicated region**	16q11.1-q13	16q11.2-q12.2	16q11.2-q12.1	16q11.2-q13	16q12.1-q13	16q11.2-q22.3	16q11.2-q22.1	16q12.1-q21	16q13-q22.3	16q12.1-q22.1	q22.1-23.1	16q13-q24
**Reference**	5	9*,11	11	4,7,8,10,11	6*	14	15	Present case	12	13	17	16
**Growth retardation**	-	-	1/1	4/6	NR^†^	-	+	-	+	+	+	NR
**Developmental delay/intellectual disability**	+	+	4/4	6/6	NR	+	+	+	+	+	+	NR
**Behavioral problems**	+	+	-	5/6	NR	NA^‡^	+	+	+	NR	+	NR
**Speech delay**	+	+	+	4/5	NR	NA	+	+	+	NR	+	NR
**Obesity**	-	+	NR	5/6	NR	-	+	+	NR	NR	NR	NR
**Dysmorphic features**	+	+	NR	4/6	NR	+	+	+	+	+	+	+
**Round face**	-	+	NR	1/6	NR	-	+	+	-	-	-	NR
**Dysplastic/low set ears**	+	-	NR	2/6	NR	+	-	+	-	-	+	NR
**Strabismus**	-	+	NR	1/6	NR	+	-	+	-	+	-	NR
**Hypermetropia**	-	+	NR	3/6	NR	+	-	+	-	-	-	NR
**Flat nasal bridge**	+	-	NR	3/6	NR	-	+	+	-	-	+	NR
**Bulbous nose**	-	-	NR	-	NR	-	+	+	-	-	-	NR
**Long, flat philtrum**	-	+	NR	1/6	NR	-	-	+	-	-	-	NR
**Small mouth**	+	-	NR	3/6	NR	-	-	+	-	-	-	NR
**Micrognathia**	+	-	NR	1/6	NR	-	-	+	-	+	-	NR
**Short neck**	-	+	NR	1/6	NR	-	-	+	-	-	-	NR
**Hypoplastic nipples**	-	-	NR	3/6	NR	-	-	+	-	-	-	NR
**Stubby fingers**	-	-	NR	3/6	NR	-	-	+	+	-	-	NR
**Congenital anomalies**												
**Skeletal**	+	+	NR	6/7	NR	+	+	+	+	NR	+	+
**Heart**	-	+	NR	1/1	NR	+	-	-	NR	-	-	+
**Urogenital**	-	+	NR	+/4	NR	-	-	-	NR	-	+	NR
**Neural tube defects/central nervous system**	-	+	NR	-	NR	-	-	-	NR	+	+	NR
**Recurrent infections**	+	-	NR	1/6	NR	-	NR	+	NR	NR	NR	NR
**Epilepsy**	-	+	NR	-	NR	-	-	-	NR	+	+	NR

*No clinical description.

†NR – not reported in the clinical description.

‡NA – not applicable.

The majority of published cases involve proximal 16q11-13 chromosome region (4-11). Several authors have attempted to characterize the proximal partial 16q trisomy syndrome. The genotype-phenotype correlation is hampered by the different size and position of the duplicated regions, uncertainty of breakpoint determination from banded karyotypes alone, small number of reported patients, and a lack of a detailed description of clinical phenotype. In addition, older cases have not been characterized by molecular methods, precluding a more precise characterization of breakpoint positions. However, all patients with proximal duplication who have available clinical description and appropriate age for evaluation have intellectual disability, speech delay, and various behavioral problems, including attention deficit hyperactivity disorder, aggressiveness, and autistic spectrum disorders (Table 1). Obesity and different mild skeletal anomalies, such as short and thick fingers or various foot deformities, are often present as well.

Two familial cases have been described. In the first family, two children inherited proximal inv dup ins(16)(q11.2q13q11.2) from the mosaic mother, while in the second family dup(16)(q11.2q21) was present in the father, his brother, and two sons (11). The clinical presentation in both families was mild, which explains the reproductive fitness and is consistent with the small size of duplicated euchromatic region.

Dysmorphic features associated with proximal dup16q include round face, flattened nasal bridge, dysplastic and low set ears, long philtrum, thin upper lip, relative micro/retrognathia, and short neck. Hypermetropia and strabismus are also often reported. These dysmorphic features are not present in patients with intermediate and intermediate-distal duplications, indicating that this particular pattern of features could be due to distinct dup 16q11.2-q13 phenotype.

The patient presented here has several of the findings common to other cases of proximal duplication 16q, such as speech delay, behavioral problems, obesity, stocky build, and characteristic dysmorphic features. On the other hand, she has only mild learning difficulties and at the age of 18 years she attends high school with adapted program, her speech is fluent, and communication and social skills are well developed.

Previous to our case study, only 4 patients with a duplication in the proximal-intermediate segment of 16q have been described. All duplications are quite large compared with the duplicated segment found in our patient (Figure 4). Mariner et al (12) described a 26-year-old woman with moderate intellectual disability, short stature, and autistic spectrum disorder carrying intermediate dup16q13-q22.3. Only conventional karyotyping was performed and the breakpoint positions were not accurately defined. Her facial appearance evidently differs from dup16q11.2-q13 phenotype. Gustavsson et al (13) described a 7-year-old girl with lumbosacral myelomeningocele, short stature, severe mental retardation, and epilepsy, carrying a 19.8 Mb duplication encompassing 16q12.1-q22.1 region. Hansson et al (14) reported on an even larger 26.17 Mb duplication of 16q11.2-q22.3 in a 25-month-old girl with mild dysmorphic features, ventricular septal defect, hearing loss, and skeletal defects, including postaxial polydactyly, clinodactyly, small feet, long hallux, and thoracic kyphoscoliosis. Recently, Lonardo et al (15) has described a 5.5-year old girl with a duplication of about 22.5 Mb spanning over 16q11.2-q22.1 region. She had moderate mental retardation, severe speech delay, obesity, and dysmorphic facial features, including round face, sparse eyebrows, long philtrum, and thin upper lip.

Only two pure duplications involve intermediate-distal segment of the 16q (16,17). Both patients had severe mental retardation and associated malformations, including heart defect, and urogenital and skeletal anomalies. Described dysmorphic features are clearly different from 16q11.2q13 phenotype and have resemblance to ATR-X syndrome (17). The presence of associated anomalies and severe mental disability in intermediate-distal duplication 16q suggests that this region could be responsible for severe phenotype and lethality of complete dup 16q (17).

Compared to other duplications that span over the proximal-intermediate region 16q11.2-q22, our patient has considerably milder phenotype. This could be explained by the small size of the duplicated region. The extent of the duplicated DNA region was defined by array CGH in order to delimit the chromosome abnormality in relation to the patient phenotype. The additional confirmation of the array CGH results by BAC-FISH excluded the rare possibility of the more complex rearrangement in the duplicated region (eg, partial triplication and duplication). The 9.92 Mb duplication contains approximately 125 RefSeq coding genes and transcripts (19). Several genes are plausible candidates for contributing to the patient's phenotype. Among them, SLC6A2 gene (OMIM 163970, Ch-Band: 16q12, DNA position: 55.69-55.73 Mb) (19), which encodes a norepinephrine transporter responsible for the reuptake of norepinephrine into presynaptic nerve terminals, has been associated with attention-deficit hyperactivity disorder (20,21). SLC6A2 gene has been examined in attention-deficit hyperactivity disorder because drugs that block the norepinephrine transporter are efficient in treating the disorder (22,23). This suggests that triple dosage of the gene product could have adverse effect on behavior by influencing noradrenaline homeostasis.

In addition, there are at least two genes that could be responsible for obesity that was present in our patient from birth to adulthood. The most obvious candidate is the FTO gene (OMIM 610966, Ch-Band: 16q12.2, DNA position: 53.74-54.15 Mb) (19), associated with body weight/obesity, metabolic disturbance, and diabetes mellitus type 2 (24). FTO gene is ubiquitously expressed, with relatively high expression in adrenal glands and brain, especially in the hypothalamus and pituitary. It has already been proposed as a candidate gene for obesity in patients with duplications partially overlapping the one seen in our patient (8,15,25,26). The second gene, BBS2 (OMIM 606151, Ch-Band: 16q21, DNA position: 56.52-56.55 Mb) (19) is a member of the Bardet-Biedl syndrome (BBS) gene family. The protein encoded by this gene, along with 6 other BBS proteins, forms a multiprotein BBSome complex required for ciliogenesis. Mutations in BBS gene family cause Bardet-Biedl syndrome, autosomal recessive disorder characterized by obesity, pigmentary retinopathy, polydactyly, renal malformation, and mental retardation. Association of variants in BB2 gene with the childhood and adult common obesity has been observed (27), but the effect of the increased copy number of the gene on phenotype is still unknown.

As the phenotypic abnormalities in patients with chromosomal duplication can also result from disruption of the genes localized at the borders of the duplicated region, we looked at the breakpoint positions. Proximal breakpoint lies close to SALL1 (OMIM 602218, Ch-Band: 16q12.1, DNA position: 51.17-51.18 Mb) (19). This is a transcriptional repressor involved in organogenesis that can cause Townes-Brocks syndrome (OMIM 107840), a rare autosomal dominant malformation syndrome featuring the combination of imperforate anus, triphalangeal or supernumerary thumbs, and sensorineural hearing loss. It is also associated with a phenotype similar to branchio-oto-renal syndrome (OMIM 113650) manifested by various combinations of preauricular pits and appendages, hearing impairment, branchial fistulas, or cysts and renal anomalies. Our patient did not have any of the clinical manifestations that could be associated with disruption of this gene. Distal breakpoint is close to CDH8 gene (OMIM 603008, Ch-Band: 16q22.1, DNA position: 61.69-62.07 Mb) (19). This gene encodes a type II classical cadherin from the cadherin superfamily, integral membrane proteins that mediate calcium-dependent cell-cell adhesion. This particular cadherin is expressed in brain and is putatively involved in synaptic adhesion, axon outgrowth, and guidance. The disruption of this gene could possibly lead to cognitive impairment.

In conclusion, in this report we presented the 10-year follow-up of a female patient with a unique dir dup(16)(q11.2q21)dn. The patient shows dysmorphic features, learning difficulties, mild mental retardation, and behavioral problems. The derivative chromosome was characterized by GTG and CTG banding, FISH, and the array CGH technique, in order to delineate breakpoints of the duplicated region. The small 9.92 Mb duplication is unique among similar published rare cases with different pure duplications of the 16q. Several genes within the duplicated region could be of interest for possible correlation with clinical features present in our patient. The analysis of the small number of reported patients with pure partial duplications of 16q indicates that clinical features seem to be distinctive between the proximal-intermediate and intermediate-distal regions of the long arm of chromosome 16. More studies of additional patients with detailed clinical and genomic characterization are needed to achieve an accurate map of different partial duplications of 16q that could be correlated with specific genes within the chromosomal regions of concern.
